# Twice-daily rivaroxaban after percutaneous left atrial appendage closure for atrial fibrillation

**DOI:** 10.3389/fphar.2024.1344828

**Published:** 2024-02-22

**Authors:** Yang-Qi Pan, Lu-Shen Jin, Sang Qian, Ting Jiang, Zhe-Ning Wang, Yi-Lian Chen, Yi-Xuan Qiu, Yi-Hao Wu, Jia-Yang Fu, Ling Li, Yuan-Nan Lin, Yue-Chun Li

**Affiliations:** Department of Cardiology, Second Affiliated Hospital and Yuying Children’s Hospital of Wenzhou Medical University, Wenzhou, China

**Keywords:** rivaroxaban, left atrial appendage occlusion, atrial fibrillation, bleeding events, device-related thrombosis, stroke

## Abstract

**Background and aim:** Rivaroxaban is an emerging oral anticoagulant for postoperative anticoagulation after percutaneous left atrial appendage closure (LAAC). Because a once-daily dosing regimen of rivaroxaban causes fluctuations in the drug plasma concentration, we studied the feasibility and safety of twice-daily rivaroxaban as a postoperative anticoagulation regimen for patients with atrial fibrillation (AF) undergoing LAAC.

**Methods:** This study involved patients with AF who underwent LAAC and took rivaroxaban postoperatively. A total of 326 patients who received a standard total dose (15 or 20 mg) of rivaroxaban based on their creatinine clearance rate were divided into the twice-daily (BID) rivaroxaban group (n = 208) and once-daily (QD) rivaroxaban group (n = 118) according to their anticoagulation strategy. Transesophageal echocardiography was recommended at 3–6 months postoperatively to check for device-related thrombosis (DRT). Clinical outcomes were evaluated during postoperative anticoagulation.

**Results:** The median CHA_2_DS_2_-VASc score (4 [3, 5] vs. 4 [3, 5], *p* = 0.28) and HAS-BLED score (2 [2, 3] vs. 2 [2, 3], *p* = 0.48) were not significantly different between the groups. During the anticoagulation period (4.1 ± 0.7 vs. 4.1 ± 0.9 months, *p* = 0.58), 148 (71.2%) patients in the BID group and 75 (63.6%) in the QD group underwent follow-up transesophageal echocardiography. There were no statistically significant differences between the two groups in terms of DRT (1.4% vs. 2.7%, *p* = 0.60), minor bleeding (8.2% vs. 11.0%, *p* = 0.39), thromboembolic events (1.0% vs. 0.8%, *p* = 1.00), major bleeding (0.5% vs. 0.8%, *p* = 1.00), or death.

**Conclusion:** A short course of twice-daily rivaroxaban following LAAC is a feasible alternative regimen with a low rate of major bleeding events, DRT, and thromboembolic events for patients with AF.

## 1 Introduction

Atrial fibrillation (AF) is the most prevalent sustained cardiac arrhythmia and is associated with high risks of ischemic stroke and other thromboembolic events. Because the left atrial appendage (LAA) is the origin of most atrial thrombi, percutaneous LAA closure (LAAC) has been used for thromboprophylaxis in patients with AF (Blackshear and Odell, 1996). Previous studies have evaluated once-daily dosing of rivaroxaban as a postoperative anticoagulation regimen in patients undergoing LAAC (Bösche et al., 2015; Bergmann et al., 2017; Enomoto et al., 2017; Gu et al., 2020; Zhu and Xu, 2021) and have demonstrated a close correlation between pharmacodynamic effects and pharmacokinetic profiles (Kubitza et al., 2005a; Kubitza et al., 2005b). However, the plasma concentration peaked as soon as 2–4 h after rivaroxaban administration, and Factor Xa activity and prolongation of prothrombin time returned to within 10% of baseline values at 24 h after rivaroxaban intake. In contrast, twice-daily administrations of rivaroxaban results in a more stable plasma concentration. A study revealed that patients who received a once-daily regimen exhibited a 60% decrease in trough plasma concentration and a 20% increase in maximum concentration, compared to those who received a twice-daily regimen, while maintaining an equivalent total dosage (Mueck et al., 2011). Therefore, twice-daily rivaroxaban has a smoother efficacy, and may be a better option after LAAC. This study was performed to assess the safety and efficacy of twice-daily rivaroxaban as an alternative regimen for patients undergoing LAAC.

## 2 Materials and methods

### 2.1 Study population

This study included consecutive patients who underwent successful implantation of either the Watchman (Boston Scientific, Marlborough, MA, USA) or the LAmbre (LifeTech Scientific, Shenzhen, China) at our institution between August 2018 and December 2020 and received rivaroxaban after the procedure. The indication for LAAC was nonvalvular AF with a CHA_2_DS_2_-VASc score of ≥2 in a patient deemed to be a poor candidate for long-term oral anticoagulation. The exclusion criteria were an intracardiac thrombus, a mechanical prosthetic heart valve, LAA obliteration, a left ventricular ejection fraction of <30%, heart transplantation, significant mitral valve stenosis, patent foramen ovale, and end-stage renal disease (creatinine clearance rate of <15 mL/min). Informed consent was obtained from all patients. Data collection and all study procedures were conducted according to the protocol approved by the Ethics Committee of the Second Affiliated Hospital of Wenzhou Medical University.

### 2.2 Catheter ablation and LAAC

The patients underwent either a one-stop procedure (combined CA and LAAC) or LAAC. Catheter ablation was performed as previously described (Ke et al., 2022). Antral ring electrical isolation of the left and right pulmonary veins was performed in all patients, and additional linear ablation was individualized according to requirements for persistent forms of AF or for redo ablation procedures. LAAC with the Watchman or LAmbre device was undertaken either before or after AF ablation according to the operator’s experience. The implantation procedure has been described in detail elsewhere (Sick et al., 2007; Lam, 2013). Briefly, the device was implanted under transesophageal echocardiography (TEE), LAA angiography, and fluoroscopic guidance via right femoral venous and transseptal access into the LAA. Ultrasonography examination was performed before discharge.

### 2.3 Anticoagulation and follow-up

After LAAC, the patients received rivaroxaban for anticoagulation twice daily or once daily (total dose of 15 mg or 20 mg) depending on the physicians. Rivaroxaban was routinely administered with discontinuation depending on the results of TEE, which was recommended at 3–6 months. Rivaroxaban was replaced by clopidogrel if TEE confirmed satisfactory device positioning and residual flow of <3 mm without device-related thrombosis (DRT) (defined as detection of thrombosis on the lumen side of the device). In patients without TEE imaging, the decision to discontinue anticoagulation was at the discretion of the physicians. During anticoagulation, we evaluated the incidence of thromboembolic and bleeding events according to the Munich consensus document (Tzikas et al., 2017). Thromboembolic events included transient ischemic attack, stroke, and systemic embolism. Bleeding events were classified as major bleeding or minor bleeding. Major bleeding was defined as pericardial bleeding, a ≥3 g/dL drop in hemoglobin or transfusion of two or more units of whole blood or red blood cells, hospitalization because of bleeding, requirement for additional surgery, life-threatening bleeding, or disabling bleeding (Tzikas et al., 2017). A bleeding event that did not meet the above criteria was defined as minor bleeding.

### 2.4 Statistical analysis

Continuous variables are reported as mean ± standard deviation or median [interquartile range], and categorical variables are expressed as count and percentage (%). Continuous variables were compared between groups using the *t*-test or Mann–Whitney *U* test as appropriate, and categorical variables were compared using the chi-square test or Fisher’s exact test. Analyses were performed with SPSS Version 26.0 (IBM Corp., Armonk, NY, USA). A *p*-value of <0.05 was considered statistically significant.

## 3 Results

### 3.1 Patient characteristics

**FIGURE 1:**
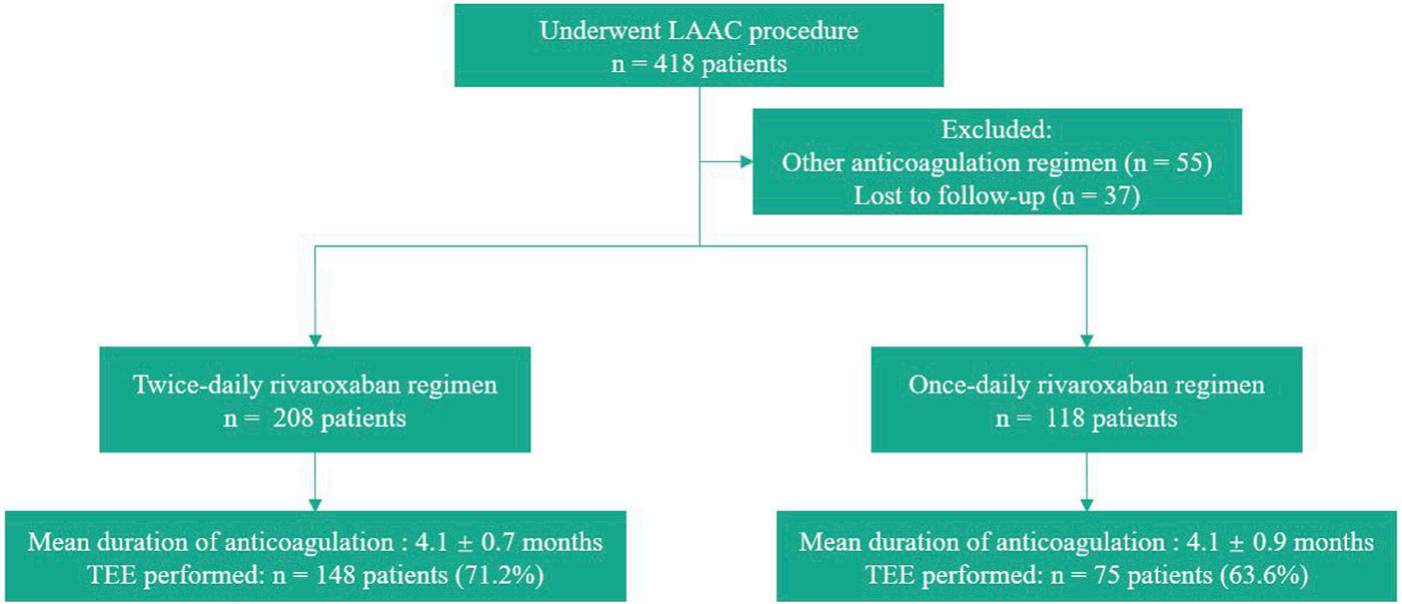
Flow Chart. LAAC, left atrial appendage closure; TEE, transesophageal echocardiography.

In total, 326 patients were included in our study and divided into two groups (Figure 1). After LAAC, 208 patients received rivaroxaban twice daily (BID group) and 118 received rivaroxaban once daily (QD group). The number of patients receiving a total dose of 20 mg was 118 in the BID group and 56 in the QD group (56.7% vs. 47.5%, *p* = 0.11). The patients’ baseline characteristics are shown in Table 1. The median CHA_2_DS_2_-VASc score (4 [3, 5] vs. 4 [3, 5], *p* = 0.28) and HAS-BLED score (2 [2, 3] vs. 2 [2, 3], *p* = 0.48) were similar between the two groups. There were no statistically significant differences in the proportion of persistent AF (65.4% vs. 63.6%, *p* = 0.74) or any other baseline characteristics between the BID and QD groups.

**TABLE 1:** Baseline characteristics of patients.

Items	BID group (n = 208)	QD group (n = 118)	*p*-Value
Age (year)	67.8 ± 8.7	67.1 ± 8.3	0.49
Female	135 (64.9%)	70 (59.3%)	0.32
CHA_2_DS_2_-VASc	4 [3,5]	4 [3,5]	0.28
HAS-BLED	2 [2,3]	2 [2,3]	0.48
Congestive heart failure	50 (24.0%)	33 (28.0%)	0.43
Hypertension	154 (74.0%)	78 (66.1%)	0.13
Diabetes mellitus	55 (26.4%)	29 (24.6%)	0.71
Previous ischemic stroke/TIA/SE	80 (38.5%)	37 (31.4%)	0.20
Prior bleeding	27 (13.0%)	10 (8.5%)	0.22
LVEF (%)	61.3 ± 9.4	61.1 ± 10.3	0.54
Creatinine clearance rate (mL/min)	76.9 [62.7,96.4]	82.4 [63.7,99.8]	0.16
Types of AF			0.74
Persistent AF	136 (65.4%)	75 (63.6%)	
Paroxysmal AF	72 (34.6%)	43 (36.4%)	
Prior interventions			
ablation	23 (11.1%)	18 (15.3%)	0.27
pacemaker	10 (4.8%)	9 (7.6%)	0.30
Total dose			0.11
20 mg	118 (56.7)	56 (47.5)	
15 mg	90 (43.3)	62 (52.5)	

Values are presented as mean ± standard deviation, n (%), or median [interquartile range]. BID, twice daily; QD, once daily; AF, atrial fibrillation; TIA, transient ischemic attack; LVEF, left ventricular ejection fraction; SE, systemic embolism.

### 3.2 Procedure and perioperative adverse events

The procedural characteristics and periprocedural adverse events are shown in Table 2. The one-stop procedure was performed in 151 (72.6%) and 85 (72.0%) patients in the BID and QD groups, respectively, while the others underwent LAAC. One major bleeding event occurred in each group (0.5% vs. 0.8%, *p* = 1.0). One patient in the BID group underwent pericardial tamponade 6 h after the combined procedure, and the patient’s hemodynamics stabilized after pericardiocentesis. One patient in the QD group developed a cerebral hemorrhage on the second postoperative day. Eight and six minor bleeding events occurred in the BID and QD groups, respectively, but did not lead to discontinuation of rivaroxaban (3.8% vs. 5.1%, *p* = 0.6). No other complications occurred during the perioperative period.

**TABLE 2:** Procedural characteristics and perioperative adverse events.

Items	BID group (n = 208)	QD group (n = 118)	*p*-Value
One-stop procedure	151 (72.6%)	85 (72.0%)	0.91
Adverse Events within the first 7 days			
Death	0	0	-
Device embolization	0	0	-
Major bleeding	1 (0.5%)	1 (0.8%)	1.00
Minor bleeding	8 (3.8%)	6 (5.1%)	0.60
Stroke, TIA or SE	0	0	-

Values are presented as n (%). BID, twice daily; QD, once daily; TIA, transient ischemic attack; SE, systemic embolism.

### 3.3 Follow-up

The follow-up results are shown in Table 3. During follow-up, 148 patients in the rivaroxaban BID group and 75 in the rivaroxaban QD group underwent postoperative TEE (71.2% vs. 63.6%, *p* = 0.16) and the mean time from the procedure to TEE was 4.2 ± 0.6 vs. 4.1 ± 1.0 months (*p* = 0.26). All patients showed residual peri-device flow of <3 mm and complete sealing of the LAA. TEE DRT was detected in two (0.7%) patients in each of the two groups by TEE (1.4% vs. 2.7%, *p* = 0.60). These four patients with a history of persistent AF were free of thromboembolic events during the follow-up period. After intensive anticoagulation, the DRT dissolved in all patients, and long-term clopidogrel was then initiated.

**TABLE 3:** Adverse events during postoperative follow-up.

Items	BID group (n = 208)	QD group (n = 118)	*p*-Value
Time of follow-up (months)	4.1 ± 0.7	4.1 ± 0.9	0.58
Death	0	0	-
Stroke, TIA or SE	2 (1.0%)	1 (0.8%)	1.00
Minor bleeding	9 (4.3%)	7 (5.9%)	0.52
Major bleeding	0	0	-
Time of TEE (months)	4.2 ± 0.6	4.0 ± 1.0	0.26
TEE performed	148 (71.2%)	75 (63.6%)	0.16
Device-related thrombosis	2 (1.4%)	2 (2.7%)	0.60
Complete sealing/peridevice flow <3 mm	148 (100%)	75 (100%)	-
Peridevice flow ≥ 3 mm	0	0	-

Values are presented as mean ± standard deviation or n (%). BID, twice daily; QD, once daily; TIA, transient ischemic attack; SE, systemic embolism; TEE, transesophageal echocardiography.

**FIGURE 2:**
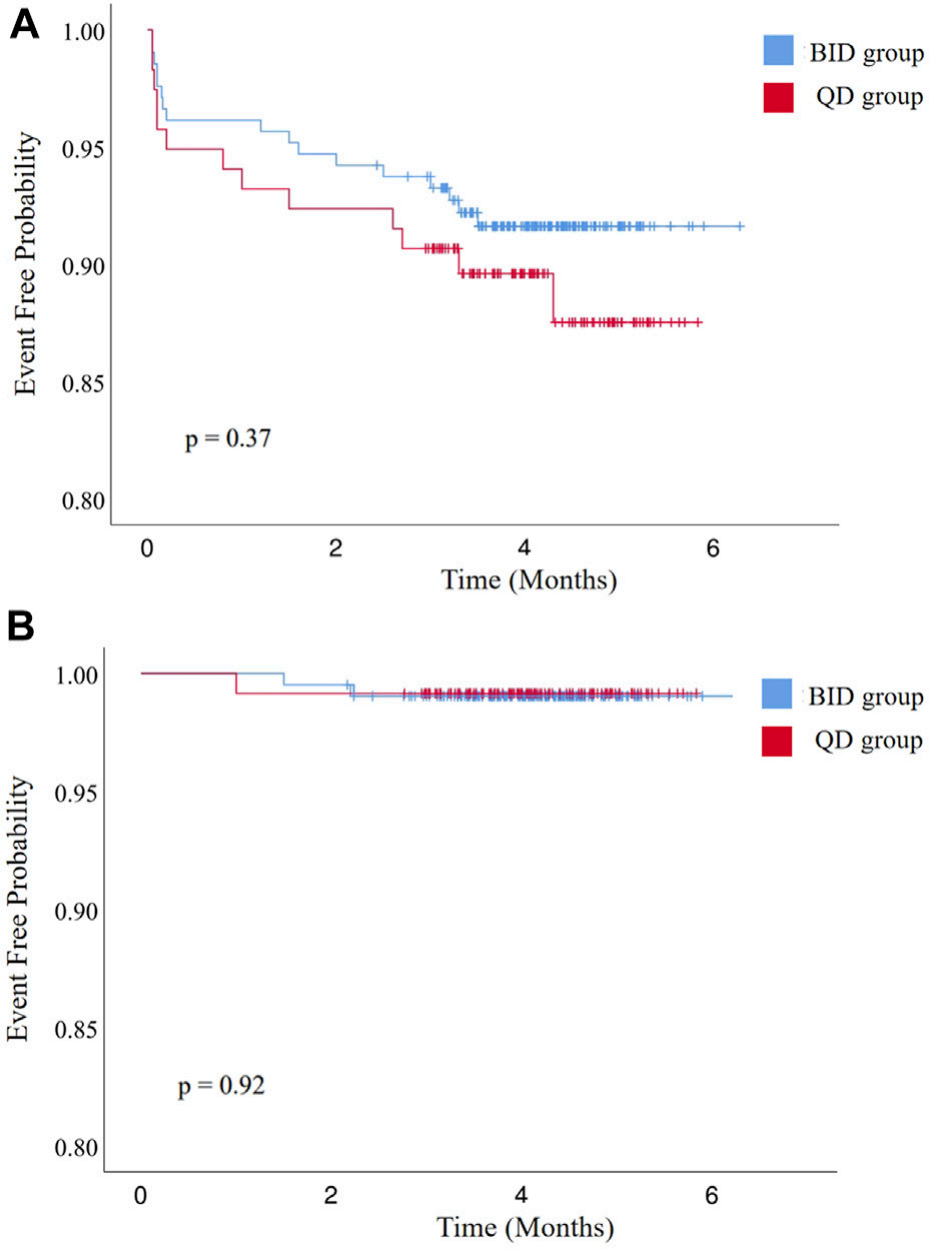
Kaplan–Meier curves of **(A)** freedom from bleeding events and **(B)** all-cause stroke, transient ischemic attack, and systemic embolism. BID, twice daily; QD, once daily.

During the postoperative anticoagulation period (4.1 ± 0.7 vs. 4.1 ± 0.9 months, *p* = 0.58), no patients died and no major bleeding events occurred (Table 3). Sixteen patients developed minor bleeding during rivaroxaban administration. Symptoms of minor bleeding events in the BID group were oral mucosal bleeding (n = 3), conjunctival congestion (n = 1), bloody stool (n = 2), hematuria (n = 1), and skin ecchymoses (n = 2). Symptoms of minor bleeding events in the QD group included oral mucosal bleeding (n = 2), hematuria (n = 1), bloody stool (n = 1), and skin ecchymoses (n = 3). Two patients receiving 10 mg rivaroxaban twice daily and one receiving 20 mg rivaroxaban once daily developed a stroke (1.0% vs. 0.8%, *p* = 1.00). During follow-up, there was no significant difference in various adverse events between the two groups (Figure 2).

## 4 Discussion

This study is the first to evaluate the feasibility and safety of taking rivaroxaban twice daily after LAAC. This medication regimen had rates of DRT, bleeding events, and thromboembolic events comparable to those of once-daily rivaroxaban, suggesting that twice-daily administration is a safe and effective postoperative antithrombotic regimen.

Rivaroxaban at 20 mg once daily has been used to prevent thrombosis and stroke after LAAC (Gu et al., 2020). The standard dose of rivaroxaban was determined by data from studies of deep vein thrombosis and exposure–response regression analysis, and subsequent studies of rivaroxaban for postoperative anticoagulation in patients undergoing LAAC followed this regimen (Mueck et al., 2011). However, the data could not reveal slight but relevant differences in major bleeding and thrombotic events between once-daily and twice-daily dosing regimens (Mainbourg et al., 2021). On the one hand, the pharmacodynamic effects of rivaroxaban are closely correlated with pharmacokinetic profiles (Kubitza et al., 2005a; Kubitza et al., 2005b). Research has shown that once-daily dosing regimens cause greater fluctuation in the rivaroxaban plasma concentration than twice-daily dosing (the maximum plasma concentration is 20% higher and the minimum is 60% lower (Mueck et al., 2011)). On the other hand, the Factor Xa activity and prolongation of prothrombin time returned to within 10% of baseline values 24 h after taking the rivaroxaban tablet (Kubitza et al., 2013). Therefore, twice-daily dosing of rivaroxaban provides better continuity of the drug plasma level than does once-daily dosing (Vrijens and Heidbuchel, 2015). In summary, compared with once-daily dosing, twice-daily dosing of rivaroxaban may be a better choice for preventing bleeding events and thrombosis after LAAC.

Twice-daily dosing of rivaroxaban has been studied in other cardiovascular diseases. A phase II trial of rivaroxaban in proximal deep vein thrombosis showed that more patients receiving rivaroxaban at 20 mg twice daily improved thrombus burden compared to patients receiving rivaroxaban once daily at the same total dose (Agnelli et al., 2007). In a randomized study of acute coronary artery disease, participants in separate groups received different total doses of rivaroxaban (5, 10, or 20 mg) by a once-daily or twice-daily regimen. The rate of clinically significant bleeding was higher in the once-daily dosing groups than in the twice-daily dosing groups, although no statistically significant difference was observed. Nevertheless, because of the lower troughs and higher peaks of plasma concentration associated with once-daily dosing, the twice-daily regimen (2.5 mg twice daily) was chosen for a phase III clinical trial and subsequently as a long-term antithrombotic treatment for secondary prevention in patients with coronary artery disease (Mega et al., 2009; Collet et al., 2020). In a noninferiority, open-label, event-driven phase II trial, 2420 patients with acute symptomatic pulmonary embolism received rivaroxaban at 15 mg twice daily for 21 days followed by 20 mg once daily. Compared with the vitamin K antagonist group, the rivaroxaban group showed noninferior primary efficacy and a reduced major bleeding rate (Kuuskne and Dankoff, 2014). However, twice-daily rivaroxaban has not been studied in patients after LAAC. Our study investigated the effectiveness and safety of a twice-daily rivaroxaban regimen in patients with AF after LAAC.

DRT is considered to develop secondary to exposure of the occluder to the circulation and is one of the main factors adversely affecting the benefit of LAAC. The PROTECT-AF study, which initially used postoperative antithrombotic therapy with warfarin, showed a DRT rate of 4.2% (20/478) (Holmes et al., 2009). In a subsequent study of DRT that analyzed 1739 patients from the PROTECT, PREVAIL, CAP, or CAP2 studies, the overall incidence of DRT was 3.7% (Dukkipati et al., 2018). The incidence of DRT after LAAC followed by non-vitamin K oral anticoagulant (NOAC) therapy in the EWOLUTION study (Bergmann et al., 2017) and in a study by Enomoto et al. (Enomoto et al., 2017) was 1.3% (1/77) and 0.9% (2/214), respectively, which was comparable to the incidence in the BID group of the present study (2/148, 1.4%) with a similar duration of follow-up. Notably, these data were obtained only for the 3-month postoperative follow-up period in the EWOLUTION study (Bergmann et al., 2017), and 60% of the patients in the study by Enomoto et al. (Enomoto et al., 2017) were followed up for only 45 days postoperatively; these factors may have resulted in a lower rate of DRT. The prospective ASAP study reported a DRT incidence of 4.0% with dual-antiplatelet therapy (DAPT) in patients contraindicated to oral anticoagulants (Reddy et al., 2013). The 3-month data from the EWOLUTION registry showed that 60.2% patients treated with DAPT had a DRT rate of 3.1% and 7.0% patients treated with single-antiplatelet therapy had a DRT rate of 3.8% (Bergmann et al., 2017), which was higher than the BID group in the present study. In our study, DRT was detected in two patients receiving the twice-daily rivaroxaban regimen and in two patients receiving the once-daily rivaroxaban regimen. Although not statistically different, the incidence of DRT was lower in the BID group than in the QD group.

The etiology of DRT remains incompletely understood. In our study, the reasons for the occurrence of DRT are unclear because most patients were highly compliant and achieved complete sealing of the LAA with a well-positioned device. Nevertheless, a study of DRT identified that DRT is closely related to a history of stroke/transient ischemic attack, a larger LAA diameter, a lower left ventricular ejection fraction, vascular disease, and permanent AF (Dukkipati et al., 2018). In addition, in two studies examining DRT, the researchers found that a dead-end structure may increase thrombogenicity and serve as a nidus for thrombus formation (Sedaghat et al., 2017; Pracon et al., 2018). Intraprocedural three-dimensional echocardiography or cardiac computed tomography may help to achieve complete coverage of the LAA ostium (Wunderlich et al., 2015; Wang et al., 2016). Moreover, close clinical follow-up and echocardiography should be performed for patients with a high risk of DRT.

Bleeding risk is inevitable with postoperative anticoagulation. A rational regimen for LAAC requires a balance between prevention of thromboembolic events and reduction of bleeding events. Several clinical studies have explored the safety of NOAC therapy after LAAC. The EWOLUTION study showed that the rate of major bleeding was lower in patients receiving NOACs (1.9%) than in those receiving warfarin (2.0%) (Bergmann et al., 2017). In a retrospective study by Zhu and Xu (Zhu and Xu, 2021), although the rates of major bleeding and thromboembolic events were similar between the NOAC and warfarin groups, the patients in the NOAC group had a significantly lower rate of minor bleeding events (5% vs. 30%, *p* < 0.01). In addition, Gu et al. (Gu et al., 2020) explored the safety and efficacy of rivaroxaban after LAAC, and Bösche et al. (Bösche et al., 2015) compared the effectiveness of NOACs with DAPT. However, the event rates in both studies were low because of the small number of cases and the short follow-up period (Bösche et al., 2015; Gu et al., 2020). Regarding antiplatelet regimens, DAPT was found to have a 2.3-fold increase in bleeding events compared to NOAC in the real-world study, while single-antiplatelet therapy had a 4.4-fold, although this was influenced by patient selection (Bergmann et al., 2017). In the study by Reddy et al., DAPT had an estimated annual bleeding rate of 6.6% (Reddy et al., 2013). In our study, the rate of all bleeding events was lower in the BID than QD group (8.6% vs. 11.9%, *p* = 0.37), but not significantly. The rate of major bleeding events in the rivaroxaban BID group was lower than in previous studies but was not significantly different from that in the QD group (0.5% vs. 0.8%, *p* = 1.00). Notably, the patient with major bleeding in the BID group of our study had undergone the one-stop procedure, and the major bleeding event was mainly attributed to operative injury caused by catheter ablation. By contrast, the patient in the QD group developed cerebral hemorrhage. Compared with serious adverse events such as stroke and major bleeding, minor bleeding events are not critical to the benefit of procedure. A few studies have documented them and there is a wide variation in incidence rates. Nonetheless, the rate of minor bleeding event in the BID group in this study was lower than the QD group without a significant difference (8.2% vs. 11.0%, *p* = 0.39). These findings suggest potentially better effects of twice-daily rivaroxaban in terms of reducing the incidence of bleeding events.

Except for percutaneous LAAC, other devices such as Lariat (SentreHEART, Inc., Redwood City, CA) and AtriClip (AtriCure, Inc., West Chester, OH) are used for epicardial exclusion of LAA. Both procedures do not require postoperative anticoagulant medications due to the absence of intracardiac devices and can be performed with acceptable rates of adverse events and success. However, both procedures are considered more complicated than LAAC and require general anesthesia. LAAC is more appealing to patients, especially those without concurrent cardiac surgery (Di Biase et al., 2024).

## 5 Limitations

The main limitations of this study are its single-center retrospective nature, limited sample size, and relatively low event rates. Another limitation is that our study involved patients who underwent the one-stop procedure and LAAC only to investigate the effect of taking rivaroxaban twice daily, which may have affected the rates of DRT and thrombotic events. Furthermore, the incidence of DRT may have been underestimated because of the possibility of undetected small DRT and the low frequency of TEE monitoring. Finally, the Watchman and LAmbre devices were used for LAAC in this study, and the results should not be generalized to other LAAC systems. In summary, the superiority of this anticoagulation regimen needs to be confirmed by further large-sample real-world studies and randomized trials.

## 6 Conclusion

The outcomes of this study suggest that taking rivaroxaban twice daily after LAAC can prevent bleeding complications without increasing the risk of DRT and thromboembolic complications and might be a viable anticoagulation alternative for patients with AF after LAAC.

## Data Availability

The raw data supporting the conclusion of this article will be made available by the authors, without undue reservation.
